# Co-Design of a Digital Health Platform for Chronic Disease Management in Rural Settings Using a Person-Centered, Collaborative-Care Model: Protocol for a 3-Phase Mixed Methods Study

**DOI:** 10.2196/77844

**Published:** 2025-12-12

**Authors:** Amanda Ellen Young, Britt Klein, Priscilla R Prestes, Colette Joy Browning, Leo R Bell, Fadi Joseph Charchar, Christopher Hall, Matthew Toms, Huy Van Nguyen, Quanda Zhang, Lisa Nicole Fiddes, Tanya E Davison, Marissa Dickins, Tim Harrison, Shane Andrew Thomas

**Affiliations:** 1 Health Innovation and Transformation Centre Federation University Mt Helen Australia; 2 Institute of Health and Wellbeing Federation University Mt Helen Australia; 3 Institute of Innovation, Science and Sustainability Federation University Mt Helen Australia; 4 Department of Physiology University of Melbourne Melbourne Australia; 5 Department of Cardiovascular Sciences University of Leicester Leicester United Kingdom; 6 WellAware.Life Pty Ltd Melbourne Australia; 7 School of Public Health University of Queensland Herston Australia; 8 Department of Population and Quantitative Health Sciences UMass Chan Medical School Worcester, MA United States; 9 Silverchain Melbourne Australia; 10 Faculty of Art, Design and Architecture Monash University Melbourne Australia; 11 Department of Psychological Sciences School of Health Sciences Swinburne University of Technology Melbourne Australia; 12 School of Public Health and Preventive Medicine Monash University Melbourne Australia; 13 Ararat Rural City Council Ararat Australia

**Keywords:** behavior change, digital health, chronic disease, prevention, early intervention, hypertension, type II diabetes, rural health

## Abstract

**Background:**

Chronic diseases represent a significant global burden, accounting for 85% of the total disease burden in Australia. This burden is particularly pronounced in rural areas, where chronic disease rates are higher, and access to health care services is more limited. Digital technology has the potential to address these disparities by overcoming challenges such as workforce shortages and geographic isolation.

**Objective:**

Our objective is to develop a digital health platform (DHP) to support the monitoring and management of chronic disease in collaboration with rural and regional stakeholders, including researchers, health care providers, and patients. The platform is being designed to be flexible, enabling applications across a range of chronic health conditions relevant to rural contexts.

**Methods:**

Guided by implementation science methodologies, we are adopting an evidence-based approach to developing a DHP for chronic disease management. Informed by co-design frameworks and best-practice guidelines, our development plan comprises three key phases: (1) stakeholder needs analysis, (2) co-design and platform development, and (3) postdesign evaluation and testing. The Federation University Human Research Ethics Committee (HREC Ref: 2023/169) granted ethics approval for this study.

**Results:**

Data collection is underway. The phase 1 review has been completed, and we have 84 survey responses. Phase 2 has commenced, with 9 workshops and 2 interviews conducted to date. Phase 3 will not commence until phase 2 has been completed. At this stage, project completion is anticipated by late 2026.

**Conclusions:**

Findings will inform the desirability, feasibility, and acceptability of co-designed DHPs for chronic disease management in rural Australia. Further, the study will contribute to the evidence base on collaborative, context-sensitive digital health innovation for resource-limited populations.

**International Registered Report Identifier (IRRID):**

DERR1-10.2196/77844

## Introduction

### Background

Chronic diseases, also known as “noncommunicable diseases” or “long-term health conditions,” are generally characterized by their persistent effects [1] and are a leading global health concern, responsible for serious illness, disability, and premature death [2]. The World Health Organization (WHO) reports that in 2020, noncommunicable diseases accounted for 7 of the world’s top 10 causes of death [3]. In Australia, recent figures indicate that chronic diseases account for 85% of the total disease burden [1]. Furthermore, approximately 90% of Australia’s nonfatal burden of disease is due to chronic conditions, with the nonfatal burden rising by 64% between 2003 and 2023 [4]. Collectively, these statistics underscore an escalating public health challenge and signal the value of pursuing effective strategies for chronic disease management and mitigation.

The increasing prevalence of chronic conditions highlights the need for innovative management strategies. Digital health solutions, including mobile apps, messaging platforms, telehealth services, wearable monitoring devices, artificial intelligence for risk prediction and precision medicine, and electronic prescribing, have the potential to enhance chronic disease management. They do so by enabling remote monitoring, personalized interventions, and greater patient engagement [5]. This is especially true in rural areas, where chronic disease rates are higher [6], and digital technology has the potential to bridge gaps caused by health care provider (HCP) shortages and geographic barriers [7].

The WHO’s Global Strategy on Digital Health 2020-2025 highlights the global momentum toward the integration of digital technologies into health care with the aim of enhancing service delivery and patient outcomes [8]. Digital health platforms (DHPs) that incorporate a range of digital health approaches can serve as a central hub, connecting systems to enable faster, more efficient, and more reliable information exchange [9]. By integrating data across various devices, these platforms enhance accessibility and improve coordination in health care delivery. However, they are often not used to their full potential due to low end-user adoption and engagement [10]. A key reason for this is thought to be the historical lack of, or limited use of, user-centric design processes in the development of digital health products [11,12]. As such, it can be argued that not involving users in the design process has led to “difficult to use” digital products that are not optimally based on end-user preference or need.

Using participatory approaches when designing digital health products provides a clearer understanding of the needs and requirements of the target populations, as well as the barriers and facilitators influencing their continued engagement with digital devices [13,14]. Recent reviews highlight recurring facilitators of digital health adoption, including improved patient-provider communication, enhanced engagement and adherence, the tailoring of tools to user needs, simplicity of use, and intuitive design [15-18]. Common barriers include limited digital literacy [19], concerns about data privacy and security [16], poor interoperability with existing systems [20], and patient preferences for face-to-face care [16]. Structural challenges such as patchy infrastructure [20], workforce issues including training and organizational support [21], and a lack of sustainable funding also constrain long-term uptake [22,23]. While these themes are well established, there is limited translation into actionable, locally grounded design. Our study addresses this gap by using co-design methods to adapt digital health solutions to the specific contexts and constraints of Australian rural populations, with the expectation that insights generated here may inform approaches in other resource-limited populations.

Findings from primary research and systematic reviews of health service co-design [24-27] suggest several key considerations relevant to the management of chronic diseases. These include involving patients, HCPs, and their service organizations from the beginning of the design process; using a variety of co-design activities (eg, workshops and interviews); involving diverse participants (eg, patients and relatives, HCPs and managers, and individuals with varying technological capabilities and application interests); prototyping and conducting iterative usability testing sessions to gather feedback; eliminating critical errors; and ongoing optimization activities.

Co-design means that user characteristics directly inform appropriate and accessible technology development. However, a recent review of the use of co-design approaches in digital health interventions found methodological problems in the conduct and reporting of these approaches and a lack of guidelines for co-design activities [28]. Beyond co-design and critical error testing phases, improvements (optimization) should continue to occur over the lifespan of a DHP (eg, acceptability or feasibility testing, efficacy testing, and implementation and beyond) [29]. To produce a needs-based, usable, engaging, and secure DHP ready for the implementation trial, it is desirable to codevelop a DHP specification document (ie, detailing the necessary digital tools, design features, and functionality required), build the digital platform, and test for critical usability errors, as well as test platform security properties (ie, penetration testing).

Rural and regional health care systems are characterized by persistent shortages of general practitioners, allied health professionals, and specialist services, alongside digital infrastructure gaps and geographical barriers to accessing care [6,30]. These constraints contribute to even further fragmented service delivery and reduced continuity of care. It can be argued that a DHP has the potential to alleviate some of these problems, as it enables the integration of multiple functions, including risk assessment, communication, patient education, and monitoring, within a single system that can be tailored to local needs. By enabling service integration across distances and supporting task-sharing among a stretched workforce, digital platforms offer a scalable and sustainable approach for improving chronic disease management in rural settings.

Despite growing recognition of the burden of chronic disease and the promise of DHPs, a notable lack of rigorously co-designed solutions tailored to rural populations remains. This study addresses this gap through a 3-phase process of needs analysis, co-design, and iterative testing, with the aim of producing a usable, acceptable, and secure platform that is grounded in end-user perspectives and optimized for real-world implementation.

### Objective

Our primary objective is to develop a specification for a DHP that supports HCPs and their patients in rural and regional settings, with the aim of facilitating the monitoring and management of chronic diseases. The platform is intended to be broadly applicable across chronic health conditions, with particular emphasis on cardiovascular disease and diabetes, reflecting their high burden in rural communities [31-34]. As such, the co-design process will engage a diverse array of participants, including patients with a variety of chronic conditions and different types of HCPs, so as to support broad applicability across chronic disease types and health care settings.

The study will use a mixed method approach, working with both HCPs and patients (1) to identify the barriers and facilitators of digital tools use as well as user preferences and needs regarding DHP features (through a literature review and survey); (2) to guide the design and refinement of the platform by evaluating potential features and overall functionality, drawing on insights gathered through participatory workshops and in-depth interviews; and (3) to detect and resolve errors in the digital platform prototype (through critical error testing).

By following a 3-phase process, comprising a needs analysis, co-design build, and postdesign development and testing, all of which incorporate direct input from community members (HCPs and patients), the co-design of the DHP will enhance the relevance and usability of health care technology. By integrating diverse perspectives, we will create a system that is user-centered and focused on end-user accessibility, efficiency, and health outcomes. This collaborative model fosters trust, improves adoption rates, and helps ensure that the platform effectively addresses real-world health care challenges [28]. Ultimately, we expect the co-design process to pave the way for a more inclusive, equitable, and impactful digital health product.

## Methods

This protocol was prepared in accordance with the SPIRIT (Standard Protocol Items: Recommendations for Interventional Trials) guidelines, and a completed SPIRIT checklist is provided as Multimedia Appendix 1.

### Setting

The target research setting is rural and regional Victoria, Australia, where health services face persistent challenges, including shortages of general practitioners, allied health professionals, and specialists [35], as well as significant travel distances and transport barriers for patients [36]. Service delivery is further constrained by variable digital infrastructure [37] and by digital literacy levels, which tend to be lower than those observed in urban areas [38]. This setting provides a critical context for developing a DHP that is responsive to rural realities and likely adaptable to other resource-limited populations.

### Design

**Figure 1:**
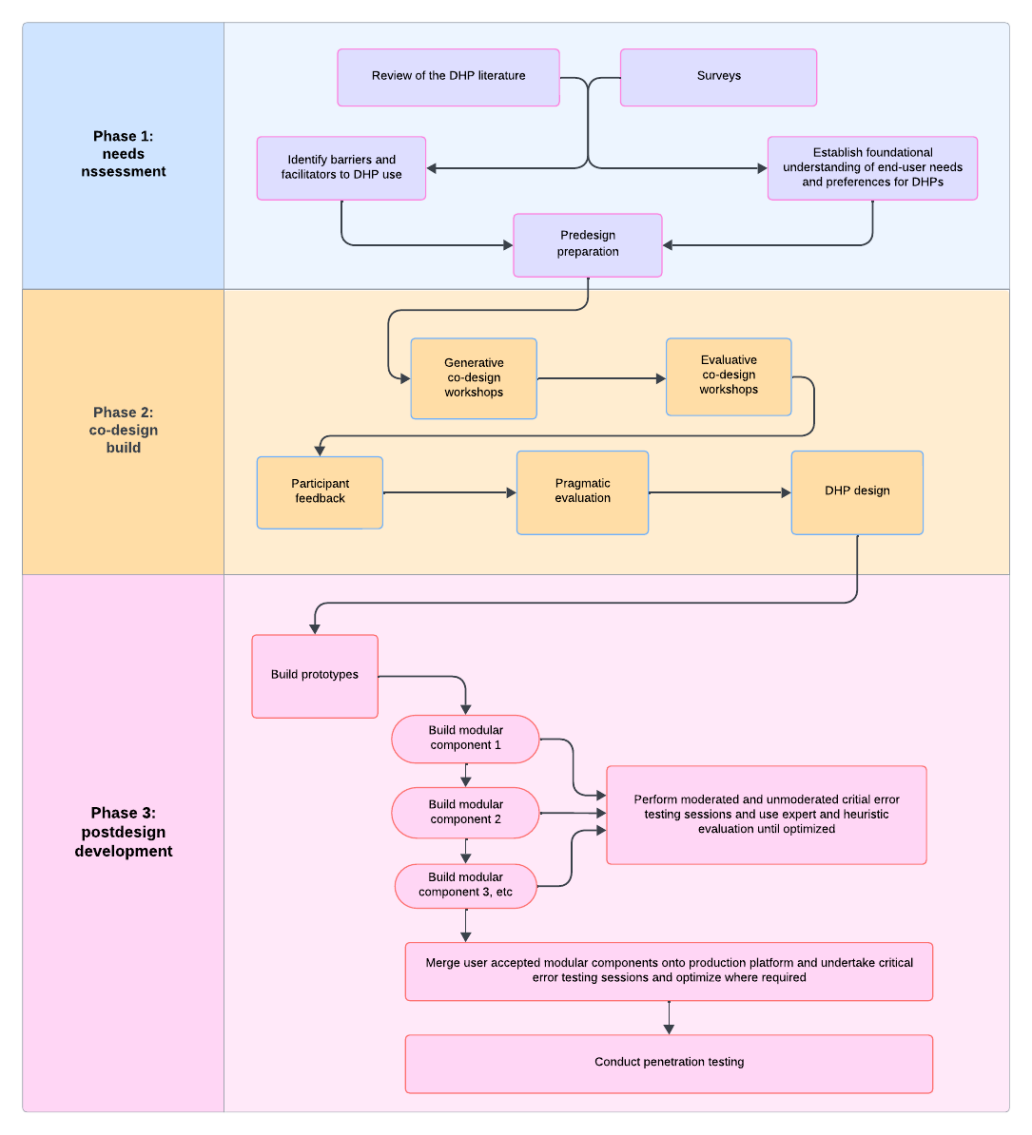
Flowchart outline of the digital cobuild phases and associated activities. DHP: digital health platform.

We will undertake the project’s “digital build” engagement in 3 phases using mixed method data collection approaches. Participants will include HCPs and health care consumers (hereafter referred to as patients) who live or work within the service areas supported by our rural and regional industry partners. The build plan includes the following components: (1) stakeholder needs analysis, (2) co-design build, and (3) postdesign development and critical error and IT security testing. A flowchart detailing the development and data collection process is provided in Figure 1, and an outline of each phase is presented in Table 1. To ensure robust theoretical foundations underpinned the project, each phase of the DHP co-design is guided by relevant frameworks and established methods (Table 1). The behavioral intervention technology model [29] is used to map the functional and technological requirements of chronic disease interventions, ensuring that both health behavior change mechanisms and platform architecture are systematically addressed. The nonadoption, abandonment, scale-up, spread, and sustainability framework [39] is applied to anticipate barriers to adoption and to consider long-term sustainability challenges, particularly relevant in rural health care contexts.

**Table 1:** Digital cobuild phases, stages, and data collection methods.

Phase and stage (sample)		Method		Frameworks or guideline and application
**1. Stakeholder needs analysis**				
	Evidence-based needs assessment (from both patient and HCPs^a^ perspectives)	A review of existing literature investigating which digital tools are the most preferred or effective when using digital platforms for chronic disease management in service delivery for HCPs and their patients.	BIT^b^ model guides the identification of functional and technological requirements for chronic disease interventions [29]. NASSS^c^ framework used prospectively to anticipate barriers to adoption and sustainability (eg, digital literacy and infrastructure in rural areas) [39].	
	Surveys (up to n=150, HCPs: n≤50 and patients: n≤100)	Online surveys, which include a series of quantitative and qualitative questions, aimed at identifying general barriers, facilitators, and preferences regarding digital tools and apps.	BIT model informs survey design by aligning questions with behavior change functions and usability factors [29]. Co-design literature emphasizes early capture of end-user perspectives to inform subsequent workshops.	
**2. Co-design**				
	Generative workshop (2 groups separately: patients+caregivers and HCPs+management)	Workshops to generate ideas regarding platform design and features required, through co-design generative activities such as storyboarding of health journey and use of personas.	Generative co-design framework for health care innovation supports structured creative exploration and ideation [40]. Extended co-design framework for mobile health results in diversity of user perspectives (different health conditions, digital literacy, and access) [41].	
	Evaluative workshop (2 groups separately: patients+caregivers and HCPs+management)	Prototypes tested with participants to confirm understanding and guide final modifications.	Agile principles and iterative prototyping continuous cycles of build-test-refine [42]. Application of user-centered design principles to validate alignment with end-user needs.	
	Interviews (HCPs and patients, where required)	Should the workshops fail to provide sufficient information for build specifications, interviews (≤ 30) will be conducted to garner greater detail or information.	Enables deeper exploration where workshops are insufficient. Guided by experience-based co-design to elicit detailed narratives [24].	
**3. Postdesign development and testing**				
	Modularly component builds (HCPs and patients)	Using agile methodologies, commence building in a modular “component” fashion. Before committing to the platform, wireframes and prototypes are developed for each modular component for feedback.	Agile or strategic modular building methods staged development with feedback loops to enhance adaptability [42].	
	Critical error testing (HCPs and patients)	Iterative usability testing of the components and the platform as a whole to make sure that all components work together.	The think-aloud protocol validated usability method capturing cognitive processes during interaction [43]. System Usability Scale standardized quantitative usability measure [44].	
	Penetration testing	A full security audit of the platform is to be undertaken by an independent third party.	Penetration tests by an ethical hacker to ensure platform robustness and compliance with digital health IT security standards [45].	

^a^HCP: health care provider.

^b^BIT: behavioral intervention technology.

^c^NASSS: nonadoption, abandonment, scale-up, spread, and sustainability.

During the co-design phases, frameworks including the generative co-design framework for health care innovation [40] and the extended co-design framework for mobile health apps [41] provide structured approaches to engaging participants with diverse perspectives and varying levels of digital literacy. These frameworks inform the design of generative and evaluative workshops, as well as semistructured interviews, ensuring that end-user input directly shapes the design features and functionality of the platform. The use of Agile methodologies [42] further supports iterative prototyping and refinement, promoting responsiveness to user feedback and practical implementation considerations.

In the postdesign testing phase, we apply well-established usability evaluation methods. The think-aloud protocol, a recognized technique in human-computer interaction research, is used to capture participants’ real-time cognitive processes as they navigate the platform, providing rich insights into usability barriers [43]. The System Usability Scale (SUS) [44] provides standardized quantitative measures of platform usability. SUS scores will be interpreted using established benchmarks, where scores above 68 are generally considered above average, and higher scores indicate better usability [46]. Consistent with established procedures [46], a mean score below this threshold will trigger targeted refinements. Scores in the “good to excellent” range (≥80) [47] will be taken as an indication that the platform is ready for larger-scale implementation trials. In this way, SUS results will not only provide a quantitative measure of usability but also function as a decision-making tool, signaling whether further iteration is required before moving to subsequent phases. The integration of these methods means that the platform will not only be functional and secure, but it will also be engaging and intuitive for end users.

Despite growing use of co-design in digital health innovation, prior initiatives are often limited by methodological fragmentation, inadequate stakeholder involvement, and insufficient attention to how rural or resource-limited settings shape feasibility [30,38,48]. Our approach addresses these limitations by embedding rural patients, caregivers, and HCPs across all 3 phases of the research. A needs analysis will reveal context-specific constraints and preferences; generative and evaluative workshops will facilitate the iterative process of feature tailoring; and usability testing will help ensure that the platform is intuitive, feasible, and acceptable in rural contexts. In this way, the study builds on but extends prior co-design literature by translating general design principles into actionable, locally grounded solutions for resource-limited rural populations.

### Participants

The study will include HCPs and patients from the specified setting. We will recruit HCPs from various discipline backgrounds, including general practitioners, nurses, mental health workers, physiotherapists, occupational therapists, dietitians, and other allied health workers. Similarly, we will aim to recruit patients with diverse demographics, backgrounds, health statuses, and conditions. All participants will be Australian residents aged 18 years or older, able to read and understand English and provide informed consent independently, and able to attend the nominated location onsite or online (in the case of workshops, interviews, and critical error testing).

To ensure equitable participation and inclusion of individuals with diverse literacy levels and digital access, we provide multiple format options, including printed materials, paper-and-pencil surveys with reply-paid envelopes, and in-person information sessions, to support participants less familiar with digital platforms. Phone-based participation options are also available for interviews and workshops to address technological or accessibility barriers. In addition, critical error testing will be used to capture perspectives across the digital-literacy spectrum, identifying potential barriers and usability issues for vulnerable groups engaging with digital health services. By including participants with limited digital access and lower levels of digital confidence, we aim to ensure that the platform features are responsive to the needs and capabilities of all end users.

### Procedure

#### Phase 1: Needs Analysis

To gain an initial understanding of barriers and facilitators of digital health tools and end-user DHP needs and preferences, we will conduct a brief environmental scan and mapping literature review and survey HCPs and patients. The literature review will focus on investigating which digital tools are the most preferred or effective when using chronic disease management digital platforms in service delivery for HCPs and patients. The survey will focus on gaining an understanding of barriers and facilitators of using digital tools in the provision of health care (eg, Wi-Fi or broadband issues in their catchment areas, digital readiness or literacy, and what health-related software they are using or have access to), perceived benefits and concerns when using digital tools (eg, more efficient but less personal, confidentiality or security), and preferences or needs around digital tools or features (eg, what digital tools or features they would like to have or use). The survey was developed based on the research team’s understanding of the domain, which includes experts in digital health, chronic disease, public health, psychology, information and technology, and software developers. Survey questions were informed by the literature and the social context of the study population and include both open- and closed-ended items. HCPs and patients will be invited to complete the survey anonymously online (hosted by SurveyMonkey) or via a paper-and-pencil version.

The survey will identify general barriers, facilitators, and preferences around digital tools and apps. Recruitment material will include the survey URL, where individuals will first read the Plain Language Information Statement (PLIS) and give consent by clicking a checkbox stating that “I understand and agree with the above conditions” before they are presented with survey items. Consent for the paper-and-pencil version will be implied by the return of the survey via a reply-paid envelope. The survey will take approximately 10 minutes to complete.

#### Phase 2: Co-Design Build

Two types of workshops will be conducted separately with HCPs and patients: (1) generative workshops to ascertain ideas regarding platform design and features and (2) evaluative workshops conducted using a prototype platform to determine whether any modifications, additions, or deletions are required ahead of the final build. The decision was made to separately collect data from HCPs and patients for a number of reasons, including likely differences in the lived experience, role-specific insights, power dynamics, and socially desirable responses.

Our industry technology partner WellAware, who have experience in DHP development and implementation, will lead workshops and interviews. Academic members of the research team will also participate in the workshop data collection and interpretation processes. Workshop discussions will be framed around topic questions relevant to health and the giving and receiving of health care services and support in rural areas. We will work to understand barriers to integrating digital health technologies into health care service delivery.

Up to 10 generative workshops will be conducted (5 for each cohort), each involving between 6 and 15 participants and running for up to 3 hours. It is believed that this workshop size supports active participation and balanced discussion, while the overall number is guided by the principles of information power [49,50]. This approach recognizes that sample adequacy depends on the richness of the data, the specificity of the research aim, and participant expertise. Empirical guidance further suggests that 3-6 focus groups per segment are typically sufficient to capture the majority of salient themes [51].

If, after the final workshop, the research team determines that novel perspectives remain to be captured, up to 30 semistructured, one-to-one interviews will be undertaken, each lasting up to 60 minutes. Interviews may also be conducted with individuals who are unable to attend a scheduled workshop. In some cases, workshop participants may be invited to elaborate further on their perspectives in an interview; however, most individuals will contribute through either a single workshop or a single interview.

Following the completion of each workshop or interview, the researchers involved will debrief to discuss the emergence of new insights. Data sufficiency will also be guided by the principles of information power [50], with saturation defined as the point at which additional data yield negligible substantive insights. A stop-rule will be applied: if successive workshops yield negligible insights, recruitment will cease; otherwise, additional workshops will be convened. Once sufficient data have been collected, the study team will convene to review co-design activity data and compile a list of requested design and digital tool features, along with accompanying descriptions. A triangulation procedure will be used to validate the findings. More specifically, the design and tool features list will be shared with selected workshop and interview participants for their review, feedback, and confirmation. Once accuracy is confirmed, a more pragmatic discussion will occur around what is achievable based on platform architecture, project timelines, and budgetary constraints.

People taking part in the generative workshops will be invited back to participate in the evaluative workshops. If rerecruitment from the generative workshop participants is insufficient, additional people will be recruited specifically for the evaluative workshops. For the evaluative usability workshops, we will recruit 6-12 participants per stakeholder group, per round. This target is consistent with human-computer interaction research, which demonstrates that sample sizes of approximately 12 are sufficient for identifying the majority of usability issues [52,53].

The evaluative workshops will focus on refining the functionality, usability, and acceptability of the DHP. Using the prototypes developed after the generative workshops, participant understanding will be checked, modifications made, and additions or deletions will be made to specify the final build. Participants will complete structured tasks that reflect typical use of the platform (eg, logging in, setting a health goal, and accessing educational resources). Facilitators will observe interactions and record usability issues, followed by a short group discussion to capture qualitative feedback on navigation, clarity, and perceived usefulness. The final design will then be discussed with the project chief investigators and finalized.

Once findings have been ratified, the write-up of the IT specification document will commence. This will be developed by WellAware team members, in conjunction with their software engineers, and it will detail all the platform works required. The resulting document will be reviewed by the chief investigators and lead partner representatives for their input and final approval. Development will begin once everyone has agreed to the works described in the IT specification document. This will include a series of sprints and testing sessions over the course of the build. The exact nature of this testing will be determined as part of the research process and outlined in the specification document.

#### Phase 3: Postdesign Development and Testing

Following the creation of the DHP prototype, critical error testing sessions will be conducted with HCPs and patients. Each session will consist of 1-3 participants and take up to 2 hours, depending on the discussion topics and dynamics. Sessions will involve participants testing the prototype and its components to ensure that any core usability errors are identified and fixed. A think-aloud process will be used, where a participant vocalizes each step of their actions as they use the platform [43]. Heat mapping software may also be used to provide data on where participants click, how far they scroll, and what they look at or ignore on the webpages. Following completion of the session, participants will be asked to complete the SUS [44]. Once the platform has been completed, we will conduct penetration testing, which will involve an independent company simulating cyberattacks on the platform to identify vulnerabilities that attackers could exploit [45]. Results will be fed back to the study team and remedied as needed.

If substantial usability or security issues are identified during late-stage testing, iterative refinement loops will be undertaken to address these issues prior to wider deployment. Final decisions regarding design modifications will be guided by clinical safety, feasibility within rural health care contexts, and alignment with evidence-based practice. Where necessary, adjustments to timelines will be made to ensure that only a functional, secure, and user-acceptable platform progresses to larger-scale trials.

### Recruitment

HCPs and patient participants will be recruited through our health care service delivery partner sites as well as through the general promotion of the study within the targeted communities. This will include print and social media, community presentations, and snowball sampling. Each health care partner will be provided with study advertisements (ie, posters and postcards) to display in staff areas and general patient waiting rooms, copies of the PLIS, and paper-and-pencil surveys (with reply-paid envelopes).

Study research fellows will conduct information and recruitment sessions onsite and online as required. In addition, HCPs and patients wanting to take part in a workshop, interview, or critical error testing session can indicate their interest by (1) contacting a study research fellow, (2) registering their interest following completion of the online survey where they will be redirected to a separate survey to provide their contact details (this will mean that survey data and contact details cannot be connected), (3) registering their interest by returning a reply slip via a reply-paid envelope available at our health care partner service delivery sites, and (4) registering their interest online by following a URL provided in the PLIS or posters.

To ensure diversity and representativeness, recruitment will be undertaken systematically across multiple rural health services, using purposive sampling to capture variation in professional roles, including general practitioners, nurses, allied health professionals, practice managers, and patient or caregiver demographics, including age, gender, geographic location, and service-use experiences. Participant characteristics will be tracked throughout, with recruitment strategies adjusted as needed to maintain breadth and balance across stakeholder groups. Multiple recruitment pathways will be used, including traditional study advertisements (ie, posters and postcards), plain language information statements, online registration, and reply-paid envelopes, to minimize barriers to participation and reduce exclusion of less technologically informed individuals. Transparent reporting of participant characteristics, response rates, and nonparticipation will further support evaluation of representativeness. Social desirability bias will be minimized through anonymized data collection, separating responses from identifying information and emphasizing confidentiality. During workshops and interviews, research fellows will use reflexivity practices, such as maintaining journals, to acknowledge potential influences on data interpretation and participant interactions. Finally, triangulation methods, including the combination of survey data and workshop findings, will be used to validate results and enhance credibility.

### Data Collection

Workshops, interviews, and critical error testing sessions will be conducted either face-to-face, online via Microsoft Teams, or by telephone (for interviews only). Participants will be nominally reimbursed with a US $19.59 gift card per hour of study involvement unless their participation is part of the HCPs’ paid work-related activities. All participants will provide written informed consent to participate in this study. For face-to-face study activities, signed consent forms will be obtained on the day. For study activities conducted online or by telephone, consent will be obtained ahead of the activity either online, by email, or regular mail. All workshops and interviews will be audio-, video-, or screen-recorded.

### Analyses

#### Phase 1

We aim to collect up to 150 survey responses (approximately 100 patients and 50 HCPs) across the participating health care service provider sites. This sample size is expected to yield sufficient data to identify key enablers and facilitators of digital tool adoption. Data from online surveys will be analyzed using descriptive statistics. Where relevant, bivariate or multivariable analyses using parametric or nonparametric tests will be conducted to assess group differences across outcomes. Open-ended survey responses will be analyzed using a structured descriptive approach. Coding will be guided by survey domains and informed by relevant theoretical frameworks (eg, digital readiness, usability, and implementation factors). This pragmatic approach will facilitate the identification of salient issues and perspectives to inform subsequent phases of the study, including co-design workshops and digital platform development.

#### Phase 2

Workshops and interviews will be audio-recorded, transcribed, and deidentified. Qualitative data will be subject to reflexive thematic analysis [54,55], in order to identify major themes relating to the DHP build and implementation. Members of the research team will meet regularly to review summaries, which will be generated after each data collection session. They will work collaboratively to establish consensus on the accuracy of the summaries and determine when saturation has been achieved. When it comes time to formally analyze the qualitative data, transcripts will be reviewed to support familiarization, and then coded inductively to capture meaningful units of text. Codes will be collated into categories and higher-order themes through an iterative process. In line with reflexive thematic analysis, formal interrater reliability statistics will not be calculated, as consensus-building is prioritized over numerical agreement [55]. NVivo (QSR International) will be used to support the coding process and facilitate data management.

#### Phase 3

We plan to conduct up to 10 critical error testing sessions with 1-3 participants in each session (n=10-30 participants, with 5-15 HCPs and 5-15 patients). Critical error testing is an iterative process in which any errors identified are fixed between sessions and continues until no further errors are found (saturation). Typically, 3-5 sessions are required to reach saturation [52]. Critical error testing session metrics will be used to identify and address usability issues. Quantitative evaluation metrics will include task completion rates, time-on-task measurements, error frequency analysis, success or failure ratios, user satisfaction scores, and the SUS questionnaire [44]. Using the method outlined in the Design section, SUS scores will be interpreted and used to guide iteration or readiness decisions. Researcher notes will support reporting on cobuild activities (eg, workflow mapping and workshop notes) and implementation metrics (eg, cost). In addition, they will be reviewed thematically for contextual or design-related factors that may account for quantitative outcomes.

### Governance, Data Storage, and Security

To manage any potential conflicts, we have adopted shared decision-making processes that emphasize collaboration and open communication. All project activities, including funding contributions and intellectual property arrangements, have been detailed in governance agreements, and records of relevant meetings and project updates are openly shared between partners. Roles and responsibilities have been clearly defined, with academic researchers maintaining authority over study design, data collection, analysis, and reporting, while the industry partner contributes technical expertise in platform development and implementation.

For the duration of the research, all electronic data will be placed into a secure, password-protected environment within the Research Data Store at Federation University. All service audit, research notes, and online survey data will be nonidentifiable, and semistructured interviews and workshop audio files and transcripts will be deidentified. Upon completing this research, all electronic data will be stored on a secure, password-protected virtual machine within the Research Data Store provided by Federation University.

### Ethical Considerations

The Federation University Human Research Ethics Committee (HREC Ref: 2023/169) granted ethics approval for this study. Per the approved protocol, all participants will provide written informed consent before participating in the study. Participants will be informed of the study purpose, procedures, potential risks, their right to decline or withdraw at any time, and the voluntary nature of participation. Consent will also be obtained for the recording of interviews and workshops. To protect privacy and confidentiality, identifiable information will be stored separately from research data on secure, password-protected institutional servers accessible only to authorized team members. Transcripts and survey data will be deidentified prior to analysis, and any quotations used in publications will be anonymized to ensure individuals cannot be identified. Participants will receive a small-value gift voucher (US $19.59) for their time, as approved by the ethics committee. They will be informed that this compensation does not depend on completing all study activities, and participants may withdraw without penalty. If there is a need to modify the study protocol for any reason, the change will be documented and sent for approval to the Federation University Human Research Ethics Committee.

## Results

The project was approved for funding in November 2022, and data collection is currently underway. Project status, as it relates to the 3 study phases, is as follows:

- Phase 1: At the time of writing, the environmental scan and mapping literature review exercise had been conducted, and we had received survey data from 35 HCPs and 49 patients.
- Phase 2: At the time of writing, we had conducted 9 workshops (5 patient and 4 HCPs). In addition, we had undertaken interviews with 2 HCPs, for which we had been unable to coordinate a convenient time for workshop participation. A preliminary analysis of the workshop and interview data was conducted to assess whether data saturation had been achieved. At the time of writing, the researcher’s assessment was that saturation had likely been achieved within the patient group (pending confirmation through more detailed analysis), while additional data were still required to reach saturation for the HCP group.
- Phase 3: This phase had not commenced at the time of writing.

We anticipate completing the project by the end of 2026.

## Discussion

### Overview

Engaging patients and HCPs throughout the design and development of the DHP helps ensure that the platform is relevant, user-friendly, and effective. Embedding the platform within a person-centered collaborative care model is a transformative strategy for managing chronic health conditions. Evidence supports this approach: digital programs developed with patient involvement have improved glycemic control in type 2 diabetes [56], and digitally supported person-centered models with structured telephone care have enhanced self-efficacy in people with chronic obstructive pulmonary disease and chronic heart failure [57]. This is consistent with systematic reviews highlighting the benefits of co-design in chronic condition management [58] and aligns with the WHO’s call for integrated, people-centered health services [59]. Our study demonstrates how these co-design principles can be operationalized in rural contexts to produce a locally grounded, scalable platform.

### Strengths and Limitations

To facilitate the successful integration of DHP findings into regional HCP services, we established early partnerships with regional health authorities. We also plan to introduce pilot programs to trial these solutions in real-world health care settings, allowing for adjustments based on local needs and challenges. Doing so will allow adaptation to local contexts, ensuring feasibility and sustainability. Ongoing feedback loops will be established to evaluate the impact of the DHP, allowing for continuous improvement and refinement as we move forward. Several strategies such as diverse recruitment pathways, anonymized data collection, reflexivity, and triangulation will be used to minimize selection and bias. These design features strengthen the validity of our findings, though residual limitations may remain.

The transferability of the study findings beyond rural and regional Victoria will be influenced by several contextual factors. The focus on health care service delivery partners across rural Victoria provides insights that may be generalizable to other rural and regional health care settings with similar demographic profiles, resources, and digital infrastructure (ie, internet access). However, transferability may be limited by factors unique to rural Victoria, such as travel distances, population density, and patient-to-health provider ratios. This means that findings, and the resultant DHP, may not be transferable to metropolitan, other regional, remote, or otherwise incomparably served areas.

We recognize that consensus in co-design activities is not always achievable, particularly where stakeholder priorities or preferences diverge. If substantial disagreement remains, final design decisions will be guided by clinical safety, feasibility within rural health care contexts, and alignment with evidence-based practice. Similarly, if the DHP is not accepted by the participants during testing, findings will be critically examined to understand the barriers to uptake. These insights will be used to adapt or refine the platform or, where appropriate, to modify implementation strategies to better fit local contexts. By anticipating these challenges, the study is positioned to generate valuable evidence about both the opportunities and the limits of DHP co-design in rural settings.

### Future Directions

Dissemination will be guided by a strategic plan targeting HCP service networks, local communities, and academic audiences. Findings will be shared through peer-reviewed publications, conference presentations, and accessible resources for HCPs and community members. To support integration into practice, partnerships with regional health authorities and pilot programs will enable adaptation to local contexts, ensuring that solutions remain both effective and sustainable. These activities lay the groundwork for the platform’s broader implementation and its long-term potential to transform rural chronic disease management.

### Conclusions

The co-design of the DHP represents a significant step toward enhancing the management of chronic conditions, particularly in rural and regional health care settings. The co-designed platform will be tailored to the needs of rural patients and HCPs. The strategic dissemination of the platform’s findings, combined with partnerships with regional health authorities and pilot testing, will support successful adoption and ongoing refinement in health care services. Ongoing feedback will support continuous improvement, with the potential to enhance outcomes and reduce inequities in rural chronic disease management and mitigation.

## Appendix

Multimedia Appendix 1 SPIRIT checklist.

## Abbreviations

DHP: digital health platform
HCP: health care provider
PLIS: Plain Language Information Statement
SPIRIT: Standard Protocol Items: Recommendations for Interventional Trials
SUS: System Usability Scale
WHO: World Health Organization
