# Supplementary material for: Co-Design of a Digital Health Platform for Chronic Disease Management in Rural Settings Using a Person-Centered, Collaborative-Care Model: Protocol for a 3-Phase Mixed Methods Study
Source: JMIR Res Protoc. 2025 Dec 12;14:e77844. doi: 10.2196/77844 (PMC12743241; doi:10.2196/77844)
Supplement: Multimedia Appendix 1 [file resprot_v14i1e77844_app1.pdf]

## SPIRIT 2013 Checklist – Completed for Protocol Submission JMIR #77844

| Section (Item) | Description | Reported in Manuscript (page #) |
|---|---|---|
| Title (1) | Descriptive title identifying study design, population, and intervention | Title page: Co-Design of a Digital Health Platform for Chronic Disease Management in Rural Settings: Implementation of a Person-Centered, Collaborative-Care Model (p. 1) |
| Trial registration (2a, 2b) | Identifier/registry name; WHO Trial Registration Data Set | N/A – Not registered |
| Protocol version (3) | Date and version identifier | N/A - Not registered |
| Funding (4) | Sources and types of financial, material, or other support | Funding section – Australian Government, Department of Education, Regional Research Collaboration Program (p. 25) |
| Roles and responsibilities (5a–d) | Names, affiliations, roles of protocol authors, and sponsor role | Author list & contributions – Acknowledgements (p. 25) |
| Background & rationale (6a, 6b) | Description of research question, justification | Introduction (p. 8-9) |
| Objectives (7) | Specific objectives/hypotheses | Objective section – Introduction (p. 9) |
| Trial design (8) | Description of design (e.g. RCT, qualitative, cohort) | Methods – Design: mixed-methods, 3-phase co-design and testing protocol (p. 10-12) |
| Study setting (9) | Description of study sites/settings | Methods – Setting: Rural and Regional Victoria (p. 10) |
| Eligibility criteria (10) | Inclusion/exclusion criteria | Methods – Participants (p. 12) |
| Interventions (11a–d) | Description of interventions, modifications, and adherence strategies | Methods – Co-design build & testing phases (p. 13-17) |
| Outcomes (12) | Primary & secondary outcomes, definitions, assessment timepoints | Methods – Analyses. Primary: System Usability Scale; Secondary: task completion, error frequency, qualitative thematic analysis (p. 19-21) |
| Participant timeline (13) | Time schedule of enrolment, interventions, assessments | Methods – Design (p. 10-12) + Table 1; Figure 1. Explicit dates not detailed. |
| Sample size (14) | Estimated participants, justification | Methods – Analyses. Power calculation for SUS with 150 survey responses ()p. 19) |
| Recruitment (15) | Plans for recruitment/retention | Methods – Recruitment (p. 17-18) |
| Sequence generation (16a) | Randomisation method | N/A – Not an RCT |

| | | |
|---|---|---|
| Allocation concealment (16b) | Allocation mechanism | N/A - no randomization is undertaken |
| Implementation (16c) | Who generates allocation, enrols, assigns | N/A - no allocation procedures required |
| Blinding (17a–b) | Who is blinded/how; circumstances for unblinding | N/A - participants and investigators are not blinded due to the co-design study design |
| Data collection methods (18a–b) | Plans for measurement, instruments, quality | Methods – Data Collection (p. 19) |
| Data management (19) | Data entry, coding, storage, security | Governance, Data storage & security (p. 21) |
| Statistical methods (20a–c) | Analysis plans, subgroup analyses, missing data | Methods – Analyses (p. 19-21) |
| Data monitoring (21a–b) | Composition/role of monitoring committee, interim analysis | N/A - observational protocol, no committee |
| Harms (22) | Plans for adverse event collection/management | Methods – Risk management in usability/security testing (p. 10, 17 & 23) |
| Auditing (23) | Frequency/procedures for independent auditing | N/A - no independent auditing is planned given the nature of the study |
| Research ethics approval (24) | Name of committee, reference number | Ethics approval (HREC Ref No. 2023/169) (p. 4) |
| Protocol amendments (25) | How changes communicated | Ethics section (p. 25) |
| Consent/assent (26a–c) | Plans for informed consent, additional provisions | Methods – Consent (p. 12, 14, 19 & 25) |
| Confidentiality (27) | How confidentiality maintained | Governance, Data storage & security (p. 18 & 21) |
| Declaration of interests (28) | Financial/competing interests | Conflicts of Interest (p. 26) |
| Access to data (29) | Who has final dataset access | Governance, Data storage & security (p. 21) |
| Ancillary/post-trial care (30) | Care/compensation provisions | N/A – no interventional procedures are involved that would necessitate post-trial care or compensation |
| Dissemination policy (31a–c) | Plans for communicating results to participants, public, journals | Discussion & Dissemination plan (p. 24) |
| Informed consent materials (32) | Model consent forms | Available on request |
| Biological specimens (33) | Plans for specimen collection/storage | N/A - biological specimens will not be collected |
